# Dual-tunnel endoscopic submucosal dissection for circumferential early gastric cancer involving the pylorus and duodenal bulb

**DOI:** 10.1055/a-2951-1258

**Published:** 2026-09-17

**Authors:** Cheng Guo, Zeyu Wang, Jingze Li, Meidong Xu

**Affiliations:** 1 Endoscopy CenterDepartment of Gastroenterology 66324 Shanghai East Hospital, Tongji University School of MedicineShanghaiShanghaiChina

Endoscopic submucosal dissection (ESD) is standard for early gastric cancer (EGC); however, pyloroduodenal lesions remain technically challenging due to complex anatomy. Despite reported traction-assisted ESD techniques for these sites, experience with truly circumferential pyloric lesions remains limited. 1​ 2​ ​3​ We present a case of complete circumferential pyloric EGC successfully resected en bloc using a dual-tunnel approach.

A 52-year-old woman presented with a circumferential, slightly elevated white lesion involving the pylorus and extending into the duodenal bulb, where it was clearly visualized upon retroflexion using a GIF-290 endoscope ( Fig. 1a, b ). Magnifying endoscopy revealed irregular microvessels and microsurface patterns on the gastric side, while on the duodenal side, elongated crypt openings were observed with white opaque substance (WOS) deposition ( Fig. 1c, d ). Inspired by the dual-tunnel technique for esophageal circumferential lesions, we applied it here to overcome the anatomical constraints ( Video 1 ). First, a circumferential mucosal incision was made on the duodenal side under retroflexed ( Fig. 2a ). Second, markings and mucosal incisions were performed on the gastric side ( Fig. 2b ). Third, an entry point was created along the greater curvature, and the first submucosal tunnel was dissected from the antrum toward the bulb ( Fig. 2c, d ), revealing the pyloric circular muscle (yellow arrow) and Brunner's glands (red arrow). Fourth, with traction suture assistance, a second tunnel was established along the lesser curvature ( Fig. 2e, f ).

**Fig. 1:**
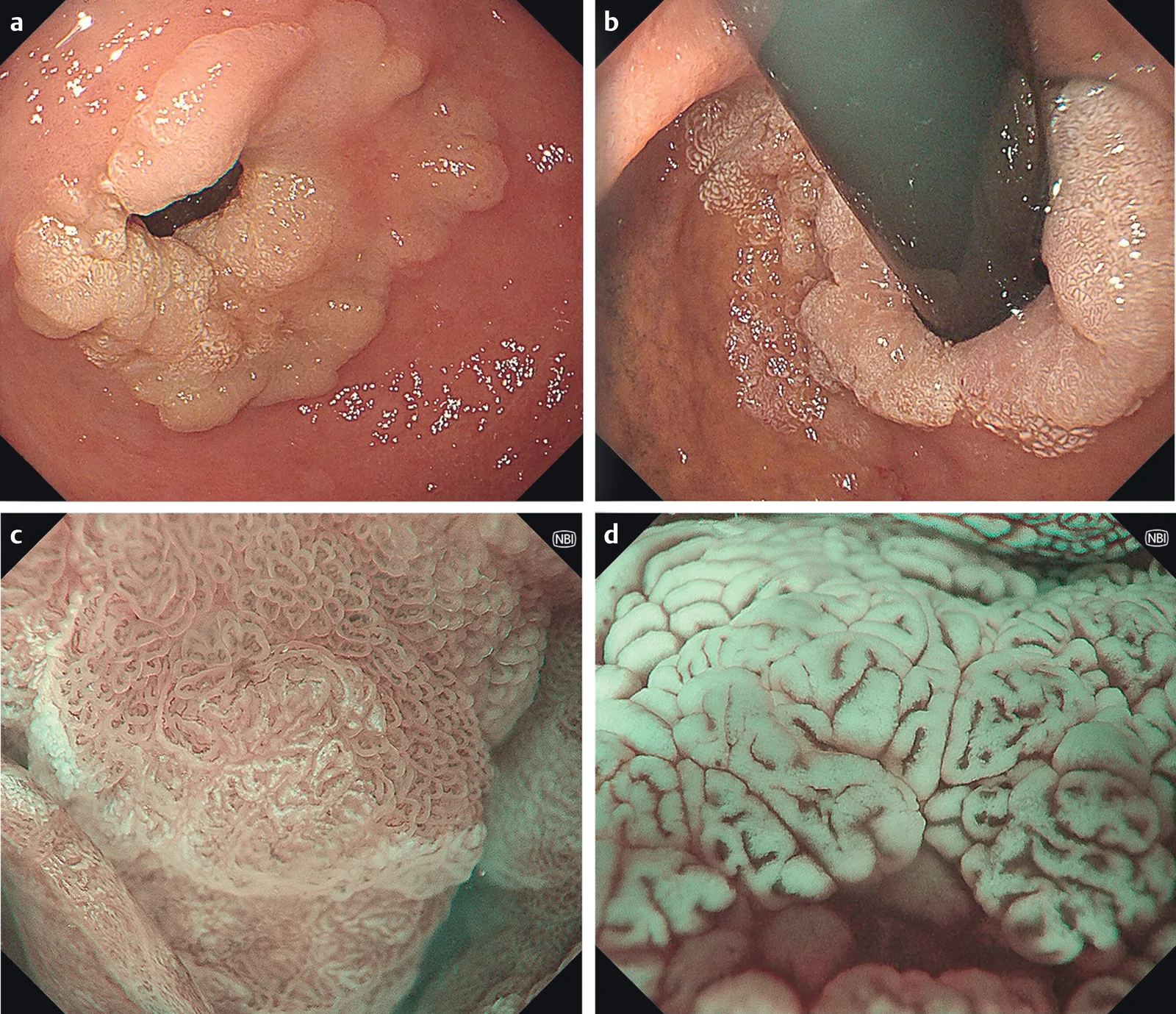
The manifestations of the lesion under white light and magnifying endoscopy. ( **a** and **b** ) The circumferential, slightly elevated white lesion involving the pylorus and extending into the duodenal bulb, where it was clearly visualized upon retroflexion using a GIF-290 endoscope. ( **c** and **d** ) The microvessels and microsurface structures on the gastric side were irregular, the crypt openings were elongated, and WOS deposits were present on the duodenal side.

**Video 1** Dual-tunnel endoscopic submucosal dissection for circumferential early gastric cancer involving the pylorus and duodenal bulb. Video URI: .


**Fig. 2:**
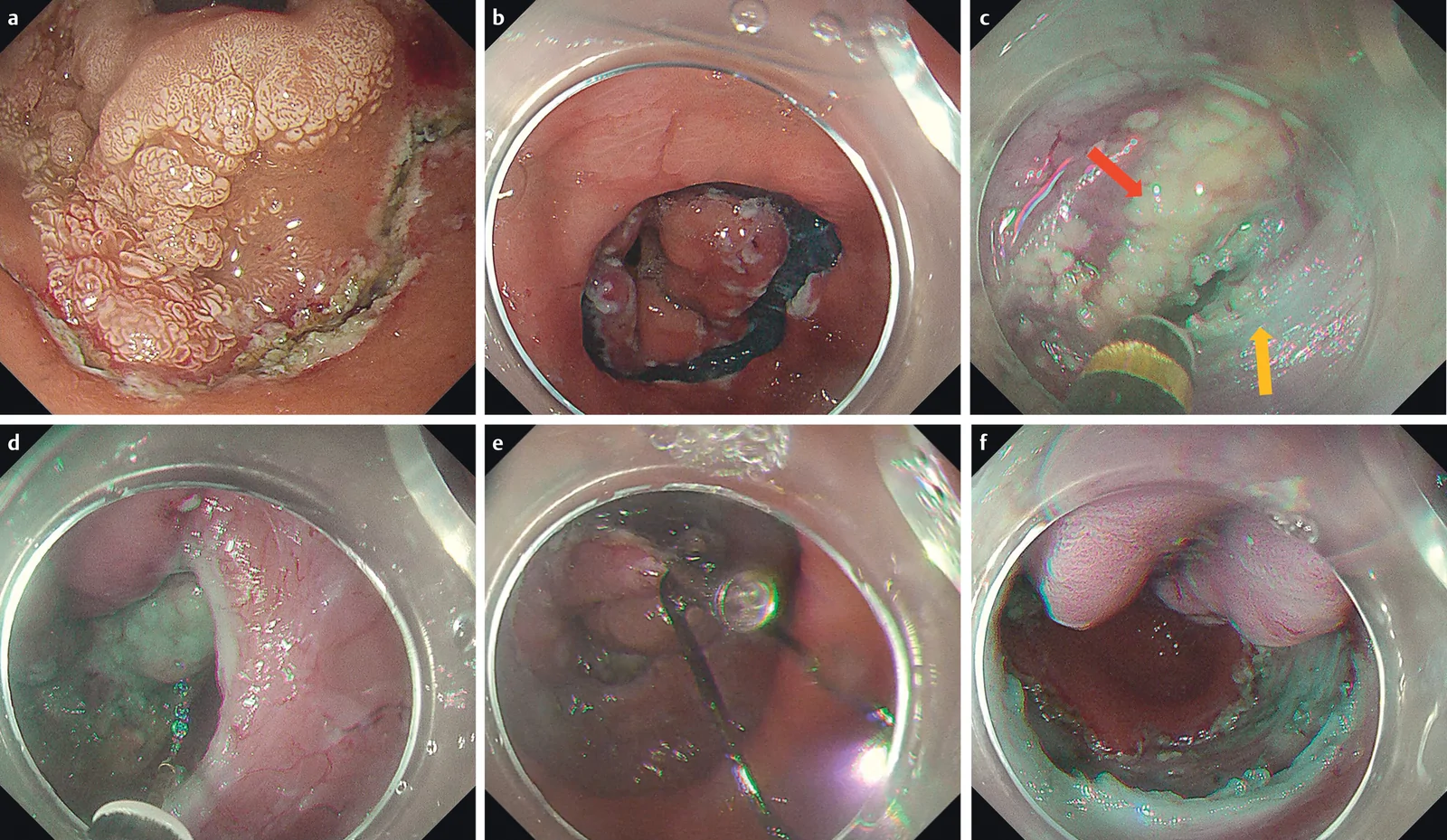
A double-tunnel ESD approach was performed for the circumferential gastric cancer. ( **a** and **b** ) Circumferential mucosal incisions were made on the duodenal and gastric side of the lesion. ( **c** and **d** ) The first submucosal tunnel was constructed from the gastric antrum toward the duodenal bulb. ( **e** and **f** ) With rubber band-assisted traction, create the second submucosal tunnel along the lesser curvature side of the lesion.

The post-dissection defect is shown; no perforation occurred ( Fig. 3a ). Histology of the 7.5×5 cm resection specimen confirmed well-differentiated tubular adenocarcinoma, classified as eCura A ( Fig. 3b ). To prevent stenosis, oral prednisolone (30 mg/d, tapered by 5 mg biweekly over 12 wk) was combined with a self-control water balloon placed on postoperative day 3. The balloon was retained for 12 weeks with regular volume adjustments, eliminating the need for repeat dilation 4​ ( Fig. 3c ). At the 5-month follow-up after the operation, the pylorus remained unobstructed, and no signs of stenosis or local recurrence were observed ( Fig. 3d ).

**Fig. 3:**
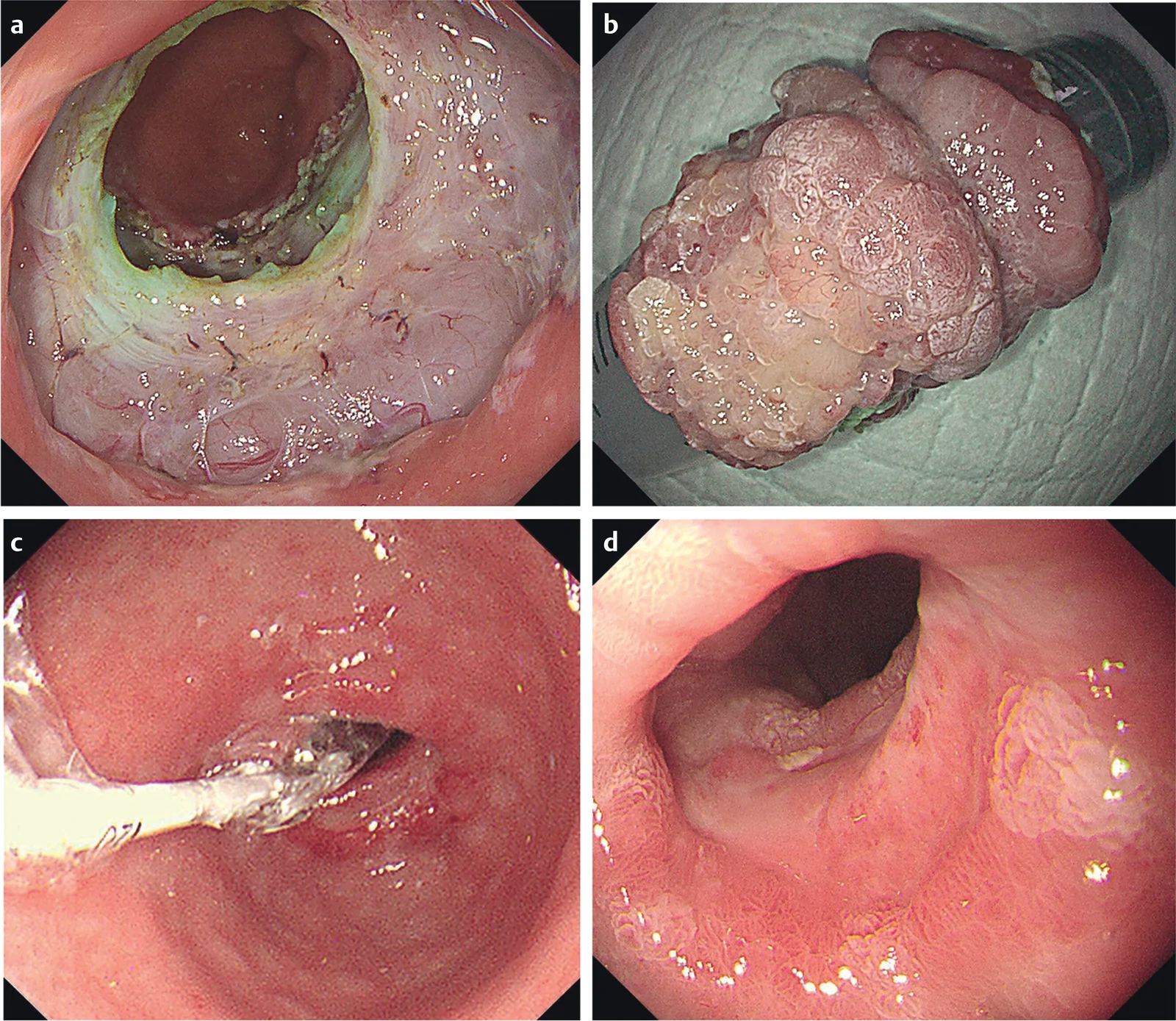
Post-ESD wound and measures to prevent stenosis. ( **a** ) The post-dissection defect is shown. ( **b** ) The resected specimen measured 7.5 × 5 cm. ( **c** ) The self-control water balloon. ( **d** ) The pylorus remained unobstructed after a 5-month follow-up period.

To our knowledge, this is the first reported en bloc ESD for a truly circumferential EGC involving the pylorus and duodenal bulb, highlighting the potential of advanced endoscopic techniques in anatomically challenging areas.

Endoscopy_UCTN_Code_TTT_1AO_2AG_3AD

## Declaration of GenAI Use

The authors used WorkBuddy (artificial intelligence [AI] writing assistant) for language polishing and grammar refinement of this manuscript. All AI-assisted contents were carefully reviewed and revised by the authors, who take full responsibility for the final content.
